# Neutrophil extracellular traps and microglia/macrophages interactions in stroke: from thromboinflammation to immunotherapy

**DOI:** 10.3389/fimmu.2026.1752471

**Published:** 2026-02-03

**Authors:** Nurittin Ardic, Rasit Dinc

**Affiliations:** 1 Med-International United Kingdom Health Agency Ltd., Nuneaton, United Kingdom; 2 INVAMED Medical Innovation Institute, New York, NY, United States

**Keywords:** immunotherapy, microglia/macrophages, neutrophil extracellular traps (NETs), stroke, thromboinflammation

## Abstract

Stroke remains a leading cause of death and disability worldwide, and inflammation is increasingly recognized as a key driver of acute injury and secondary neurodegeneration. Among post-stroke immune mediators, neutrophil extracellular traps (NETs) have emerged as critical amplifiers of thromboinflammation and cerebrovascular injury. Parallel developments highlight microglia and infiltrating macrophages as key regulators of sterile inflammation in ischemic stroke (IS), intracerebral hemorrhage (ICH), and subarachnoid hemorrhage (SAH). However, the bidirectional interaction between NETs and microglia/macrophages has not been comprehensively analyzed despite its translational importance. This review describes the mechanistic pathways by which NET components activate microglial pattern recognition receptors, triggering inflammasome activation, inflammatory signaling cascades, and cytokine release. Activated microglia, in turn, promote neutrophil recruitment and NETosis, creating a self-reinforcing cycle. Evidence from ischemic and hemorrhagic stroke demonstrates how NET-microglia interactions lead to neurovascular complications such as blood-brain barrier disruption, microvascular dysfunction, and neuronal injury. We examine therapeutic strategies targeting NET formation and destruction, microglial modulation, and combination approaches to interrupt this inflammatory axis. We highlight novel biomarker and imaging approaches that may enable personalized immunotherapy. Together, these strategies position the NET-microglia/macrophage axis as a promising immunomodulatory target in ischemic and hemorrhagic stroke, offering new avenues for precision therapy development.

## Graphical Abstract

**Figure:**
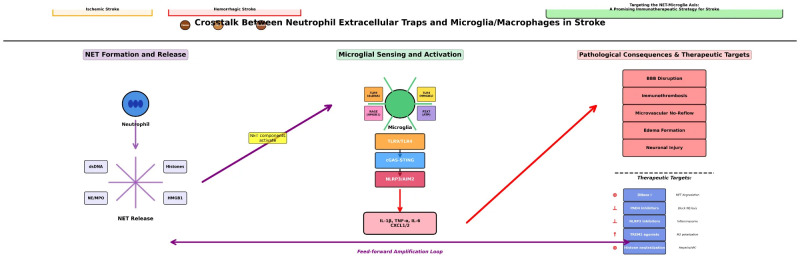
This study provides an overview of the bidirectional interaction between neutrophil extracellular traps (NETs) and microglia/macrophages in ischemic and hemorrhagic stroke, demonstrating how feedforward inflammatory signaling links intravascular immunothrombosis to neurovascular damage and highlighting important opportunities for therapeutic intervention along this axis.

## 1 Introduction

Stroke is the second leading cause of death worldwide and contributes significantly to disability (1). Ischemic stroke (IS) accounts for approximately 80-85% of cases, while intracerebral or subarachnoid hemorrhage (ICH/SAH) represents the remainder (2, 3). Despite significant advances in reperfusion therapies, including intravenous thrombolysis and mechanical thrombectomy, outcomes remain suboptimal for a large proportion of patients. Increasing evidence suggests that, beyond vascular occlusion and neuronal ischemia, immune-mediated thromboinflammation is a central determinant of both acute injury and secondary neurodegeneration (4, 5). Within this framework, neutrophils and microglia/macrophages, the key innate immune cells of the brain and its periphery, have emerged as critical regulators of the inflammatory milieu that shapes tissue fate.

Neutrophils are among the earliest responders to cerebrovascular injury, infiltrating ischemic or hemorrhagic areas within minutes to hours. One of their most potent mechanisms of action is the release of neutrophil extracellular traps (NETs), chromatin-based structures containing histones, proteases, and antimicrobial proteins (described in detail in Section 2) (6). While NETs contribute to host defense, their excessive or sustained formation in sterile inflammations such as stroke promotes microvascular thrombosis, blood-brain barrier (BBB) disruption, endothelial death, and poor collateral perfusion (7, 8). NET-rich thrombi are increasingly recognized in human stroke specimens and are associated with resistance to mechanical thrombectomy and decreased response to tissue plasminogen activator (9).

Microglia, the resident macrophages of the central nervous system (CNS), and infiltrating monocyte-derived macrophages are the primary immune responses within the brain parenchyma after stroke. These cells exhibit remarkable phenotypic and functional plasticity, switching between proinflammatory, tissue-damaging states and reparative, resolving phenotypes depending on microenvironmental cues (10, 11). Activated microglia rapidly detect danger-associated molecular patterns (DAMPs), coordinate cytokine and chemokine release, and play a role in synaptic dehiscence, phagocytosis of cellular debris, and neuronal network remodeling. Importantly, microglial responses differ markedly between IS, ICH, and SAH, reflecting distinct injury dynamics, hematoma-induced danger signals, and cell-cell interactions (12).

Recent studies have highlighted a bidirectional interaction between NETs and microglia/macrophages that amplifies neuroinflammation and shapes stroke outcomes (11). NET-derived DNA, histones, and proteases can directly activate microglial receptors such as TLR9, cGAS–STING, TLR4, and NLRP3 inflammasome components, promoting cytokine release and pyroptotic responses (13). In turn, microglia-derived IL-1β, TNF-α, IL-8/CXCL8, and reactive oxygen species (ROS) promote further NETosis via the P2X7–NLRP3 pathway and chemotactic recruitment of circulating neutrophils (14). Thus, NET-microglia interactions create a self-amplifying inflammatory cycle that exacerbates BBB damage, enhances infarct expansion, and facilitates hematoma toxicity in ICH.

This immunological interaction is further shaped by temporal dynamics. While NET formation peaks early, typically within the first 6–24 hours after stroke onset, microglial activation evolves over days and weeks, influencing secondary damage, neuroplasticity, and long-term outcomes (4, 15). The spatial organization of these interactions (e.g., accumulation of NETs in peri-infarct microvessels and clustering of microglia around necrotic tissue) also determines the degree of local thrombo-inflammatory response and microvascular failure (5, 16). This information highlights the need to understand NET-microglia communication not as isolated events but as a coordinated and evolving innate immune signaling network.

Given these mechanistic advances, therapeutic targeting of the NET-microglia/macrophage axis has gained significant momentum. Experimental evidence supports the use of DNase I, PAD4 inhibitors, NE/MPO inhibitors, and histone neutralizing agents to reduce NET-induced pathology, while microglia-modulating strategies such as NLRP3 inhibition, CSF1R modulation, and TREM2 agonism offer complementary approaches to control damaging inflammation (17). Combination therapies that jointly target neutrophil and microglial pathways are paving the way for a new era in stroke immunotherapy. Parallel developments in NET biomarkers, thrombus tissue analysis, and molecular imaging may further accelerate clinical translation.

In this review, we present an integrated analysis of NET-microglia/macrophage interactions throughout ischemic and hemorrhagic stroke. First, we summarize NET formation, molecular composition, and functional roles in stroke pathophysiology (Sections 2-5). Next, we examine the bidirectional interaction between NETs and microglia/macrophages; focusing on how NET-derived danger signals activate microglial sensing and inflammation programs, and how microglial inflammatory outputs, in turn, enhance neutrophil recruitment and NETosis (Sections 6-7). We then discuss novel therapeutic strategies designed to disrupt this amplification cycle, including combination and temporally phased regimens, as well as approaches that inhibit NET formation, promote NET clearance, and modulate maladaptive microglial/macrophage activation (Section 8). Finally, we review the translation opportunities and challenges, including important considerations in timing, implementation, and safety in clinical trial design, as well as biomarker and imaging approaches for patient stratification and treatment monitoring (Sections 9-10).

**Figure 1:**
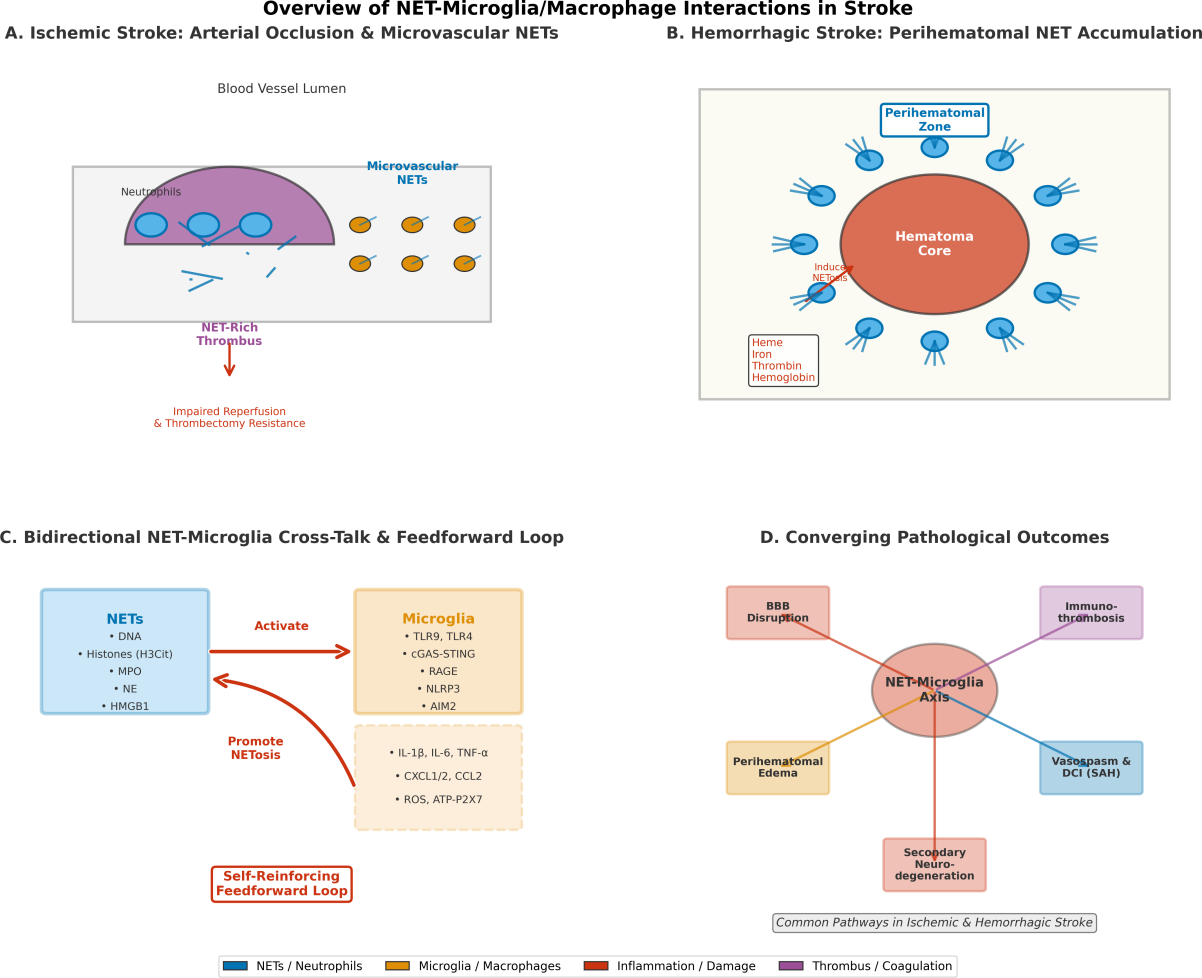
Conceptual framework of NET-microglia/macrophage interactions in stroke subtypes. Figure **(A)** illustrates NET-rich arterial thrombi and downstream microvascular occlusion in ischemic stroke, **(B)** perihematomal NET deposition and microglial activation in hemorrhagic stroke, **(C)** bidirectional molecular cross-interaction between NET components and microglial pattern recognition receptors, and **(D)** convergent pathological outcomes including blood-brain barrier disruption, immune thrombosis, and secondary neurodegeneration. Figure and color coding indicate immune cell types, NET structures, and the direction of signaling pathways.

**Figure 1** presents a unifying conceptual framework demonstrating how NET formation in both ischemic and hemorrhagic stroke is spatially dependent on microglia activation, leading to a feedforward inflammatory cycle that results in blood-brain barrier disruption, microvascular dysfunction, and secondary neurodegeneration.

## 2 Neutrophil extracellular traps: formation, structure, and functions

Neutrophil extracellular traps (NETs) are net-like, chromatin-based structures released by activated neutrophils as part of an evolutionarily conserved host defense mechanism. First identified in 2004, NETs have since been recognized as key mediators of immunothrombosis and tissue damage in various sterile inflammatory conditions, including ischemic and hemorrhagic stroke (15, 18). NETs, composed of decondensed DNA structures decorated with histones, neutrophil elastase (NE), myeloperoxidase (MPO), cathepsin G, MMP-9, and other granular proteins, serve as both antimicrobial structures and potent enhancers of inflammatory and thrombotic signaling (14, 19). In the cerebrovascular context, accumulating evidence suggests that NETs contribute significantly to microvascular obstruction, BBB damage, and secondary neuronal injury.

### 2.1 NET formation pathways: suicidal and vital NETosis

NETs can be generated through different cellular programs, broadly categorized as suicidal NETosis and vital NET release.

Suicidal NETosis is the canonical pathway driven by robust intracellular signaling, resulting in nuclear envelope disruption, chromatin decondensation, and cell lysis. This process is stimulated by signals such as phorbol 12-myristate 13-acetate (PMA), lipopolysaccharide (LPS), calcium ionophores, and inflammatory cytokines and typically progresses within hours (19, 20). A central feature is the activation of the protein arginine deiminase 4 (PAD4), which citrullinates histones, leading to chromatin decondensation and expulsion into the extracellular space (21). PAD4-dependent NETosis is strongly implicated in ischemic stroke, where genetic deletion or pharmacological inhibition of PAD4 reduces infarct size and improves microvascular perfusion (7). Beyond stroke, NETs are increasingly recognized as central mediators of inflammatory thrombosis across the cardiovascular spectrum, including venous thromboembolism and atherosclerotic disease (22, 23).

Vital NETosis, in contrast, occurs rapidly, within minutes, and preserves neutrophil viability. In this form, chromatin and granular components are expelled via vesicular trafficking without rupturing the plasma membrane (24). Vital NET release is triggered by interactions with activated platelets, complement activation (C5a), and engagement of toll-like receptors (TLRs), particularly TLR2 and TLR4. Platelet-mediated NETosis is highly relevant to stroke pathophysiology, where platelet-neutrophil aggregates accumulate in occlusive thrombi and microvessels shortly after ischemia or hemorrhage (8, 9).

### 2.2 Structure and molecular components of NETs

NETs exhibit a highly organized ultrastructure consisting of:

• Decondensed chromatin fibers (∼15–25 nm in diameter) that form the backbone
• Histones (H2A, H2B, H3, H4) containing distinctive biochemical markers such as citrullinated H3 (H3Cit)
• Granule proteins such as NE, MPO, cathepsin G, lactoferrin, and proteinase 3
• Antimicrobial peptides (LL-37, defensins)
• Matrix metalloproteinases (MMP-9)
• Complement components and tissue factor attached to NET scaffolds

These components confer potent cytotoxic, prothrombotic, and proinflammatory properties to NETs (11, 19, 23). Histones function as major DAMPs that can trigger endothelial death and increase BBB permeability, while NE and MPO disrupt the extracellular matrix and disrupt endothelial tight junctions. In stroke thrombi, NET-associated tissue factor contributes to fibrin formation and clot stabilization, increasing resistance to fibrinolysis (23).

### 2.3 Physiological and pathological roles of NETs

Physiologically, NETs capture pathogens and prevent local dissemination during infection, contributing to the protection of the innate immune system (18, 25). However, excessive, or unregulated NET formation triggers pathological processes, particularly in the vasculature. In stroke, NETs promote immunothrombosis, defined as the convergence of immune and coagulation pathways to create intravascular occlusions (26). NET scaffolds increase platelet adhesion, activate factor XII, and accelerate fibrin deposition, ultimately contributing to the microvascular “no-backflow” phenomenon after recanalization treatments (7). NETs also act as upstream activators of microglia, astrocytes, and endothelial cells, increasing local inflammation. NET-derived DNA and histones activate the TLR9, TLR4, or cGAS-STING pathways in innate immune cells, while NET proteases degrade the extracellular matrix and compromise BBB integrity (13). Collectively, these effects exacerbate neuronal death, infarct expansion, and perihematomal damage. Importantly, the persistence of NETs beyond the acute phase may impede neurovascular repair by impairing angiogenesis and promoting chronic microglial activation (11). These findings are also paralleled in other vascular beds where NET-induced thrombosis has been described (26).

### 2.4 Mechanisms of NET degradation and clearance

NETs are normally cleared by serum nucleases (predominantly DNase I and DNase1L3) and phagocytes, including macrophages and microglia, which can engulf NET fragments and degrade them via the lysosomal pathway (19, 26). However, during stroke, NET clearance is frequently impaired due to hypoxia, oxidative stress, and inflammatory microenvironments, leading to NET accumulation in both microvasculature and parenchyma. Decreased NET clearance has been associated with larger infarcts, higher serum NET biomarkers, and worse neurological outcomes in clinical cohorts (16, 27). Therefore, therapeutically enhancing NET degradation, particularly with exogenous DNase I, is a promising strategy that will be explored in the following sections.

## 3 Microglia and macrophages in stroke

Microglia, the resident immune cells of the central nervous system (CNS), and infiltrating monocyte-derived macrophages constitute the primary phagocytic cells and inflammatory mediators that shape the brain’s response to ischemic and hemorrhagic injury. Their activation is rapid, dynamic, and context-dependent, reflecting both the initial vascular injury and the evolving microenvironment of the injured tissue (10, 22, 26). While microglia maintain tissue surveillance and synaptic homeostasis under physiological conditions, stroke triggers a profound reprogramming toward inflammatory, phagocytic, or reparative states that critically impact neuronal survival, edema formation, and long-term recovery.

### 3.1 Microglia: resident macrophages with rapid response ability

Microglia originate from yolk sac precursor cells and are uniquely adapted to detect changes in neuronal and vascular homeostasis. After stroke onset, microglia undergo a morphological transformation from ramified surveillance cells to hypertrophic or amoeboid-activated forms within minutes to hours (4, 12). This early activation is driven by damage-associated molecular patterns (DAMPs), such as ATP, HMGB1, extracellular DNA, heme, and oxidized lipids, released from damaged neurons, endothelial cells, or, in the case of hemorrhage, red blood cells. Activated microglia activate various pattern recognition receptors, including TLR2, TLR4, TLR9, NOD-like receptors, and the cGAS-STING pathway, initiating intracellular signaling cascades that result in cytokine production (IL-1β, IL-6, TNF-α), chemokine secretion (CCL2, CXCL1/CXCL2), and inflammasome activation (NLRP3, AIM2) (27, 28). These early microglial responses shape the extent of acute injury by regulating neutrophil recruitment, BBB permeability, and local thrombogenic signaling.

### 3.2 Infiltrating macrophages: monocytes crossing the compromised blood-brain barrier

While microglia play a dominant role in the acute response, peripheral monocytes begin to infiltrate the brain 12–24 hours after stroke, peaking around days 3–5, particularly in ischemic cores and perihematomal regions (4, 12, 15). This infiltration is dependent on chemokine gradients, particularly CCL2-CCR2 interactions, and is significantly increased when the BBB is damaged by NETs, thrombin, hemoglobin metabolites, or inflammatory cytokines. Monocyte-derived macrophages contribute to debris clearance, hematoma resolution, and tissue remodeling, but can also propagate inflammation, particularly through the sustained production of IL-1β, ROS, and matrix metalloproteinases (MMP-9) (8, 17). Their relative balance with microglia (reinforced by local cytokines and NET-derived components) plays a crucial role in determining whether damaged tissue progresses toward repair or secondary degeneration.

### 3.3 Phenotypic plasticity: beyond the M1/M2 paradigm

Microglia and macrophages exhibit remarkable functional flexibility. While conventional nomenclature divides them into M1-like (pro-inflammatory) and M2-like (anti-inflammatory, reparative) phenotypes, single-cell transcriptomic studies reveal much greater heterogeneity, including disease-associated microglia (DAM), lipid droplet-accumulating microglia, interferon-responsive states, and post-hemorrhage heme processing phenotypes (29, 30).

Pro-inflammatory states are characterized by NLRP3 activation, IL-1β and TNF-α release, nitric oxide synthesis, and promotion of leukocyte recruitment.

Reparative states involve upregulation of TREM2, Arg1, Ym1, and genes associated with phagocytosis, angiogenesis, and extracellular matrix remodeling. Stroke dynamically shifts microglia/macrophages along these spectrums. In early ischemia, inflammatory phenotypes driven by DAMPs, NET DNA, histones, and ROS predominate (31, 32). Later stages support reparative microglia that contribute to synaptic pruning, hematoma clearance, white matter support, and neurovascular recovery. However, persistent NET-rich microenvironments, particularly at the microvascular interface, may prevent this transition, locking microglia into detrimental inflammatory states (5, 12).

### 3.4 Roles of microglia and macrophages in ischemic stroke

In ischemic stroke, microglia rapidly accumulate in peri-infarct regions, where they interact with endothelial cells, neurons, and infiltrating neutrophils. They promote BBB breakdown via cytokines, MMPs, and ROS as well as enhancing leukocyte recruitment via CCL2, CXCL1/2, and TNF-α. On the other hand, microglia phagocytose dead neurons, clear extracellular debris, and regulate excitotoxicity via modulation of glutamate transport (33, 34).

Microglia-neutrophil interactions are particularly important. Microglial cytokines (IL-1β, IL-6) enhance neutrophil activation, while NETs activate microglial TLR9 or NLRP3, creating a reinforcing feedback loop that accelerates infarct expansion. In late stages, microglia/macrophages facilitate angiogenesis and tissue remodeling, but maladaptive responses such as prolonged inflammasome activation can impair healing and promote chronic CNS inflammation (35, 36).

### 3.5 Microglial and macrophage roles in hemorrhagic stroke

Hemorrhagic stroke presents additional complexities due to the presence of blood-borne toxins. Microglia and macrophages play a central role in:

• Phagocytosis of erythrocytes (erythrophagocytosis)
• Heme and hemoglobin metabolism via HO-1, CD163, and ferritin
• Regulation of perihematomal edema
• Inflammasome activation by heme, iron, and NET proteins

Microglial activation in ICH and SAH is more intense and prolonged than ischemia driven by hemoglobin degradation products, lasting for days or even weeks (12, 35, 36). NETs accumulated in both hematomas and surrounding microvessels further activate microglia via the TLR4, cGAS-STING, and AIM2 pathways, contributing to neuronal death, vasospasm (in SAH), and delayed cerebral ischemia (37). Macrophage phenotypes in hemorrhage are particularly influenced by iron and heme signaling: While some promote hematoma clearance and healing, iron-induced oxidative stress can perpetuate chronic inflammation and white matter damage. Therefore, therapeutic manipulation of microglial and macrophage polarization has emerged as a promising strategy in ICH, as highlighted in our recent review on immunomodulation in hemorrhagic stroke (30).

### 3.6 Interactions with other innate and adaptive immune cells

Microglia and macrophages also regulate the broader immune response. They shape T cell recruitment and polarization, regulate astrocyte reactivity, and interact with endothelial cells to alter cerebrovascular tone and permeability. Importantly, microglial cell crosstalk with neutrophils (via cytokines, chemokines, ROS, and direct contact) forms a critical axis that determines the severity of early thromboinflammation (38). This forms the basis for Section 4, where the bidirectional interaction with NETs is examined in mechanistic detail.

## 4 NETs in ischemic stroke

Neutrophil extracellular traps (NETs) have emerged as central mediators of ischemic stroke (IS) pathophysiology, contributing to vascular occlusion, impaired reperfusion, microvascular thrombosis, BBB disruption, and secondary neurotoxicity. Experimental and clinical studies consistently demonstrate that NET accumulation begins within hours of ischemia onset and continues throughout the acute and subacute phases, shaping the course of infarct expansion and functional recovery (7, 8, 27). The pathogenic roles of NETs extend from large vessel occlusions to microcirculatory failure and tightly link innate immunity to cerebrovascular injury.

### 4.1 NETs in thrombus formation and large vessel occlusion

NETs directly contribute to the formation and stabilization of arterial thrombi in I**S**. In human mechanical thrombectomy specimens, NETs form dense scaffolds within the fibrin-platelet matrix, bind to tissue factor, activate factor XII, and enhance thrombin generation, supporting the concept of NET-induced immunothrombosis (7, 9). NET-rich thrombi are mechanically stiffer, more cohesive, and more resistant to fragmentation, making recovery more difficult and increasing the number of passes required during thrombectomy (8). Importantly, NET content is higher in cardioembolic, and large-artery atherosclerotic strokes compared to small-vessel occlusions, suggesting that NETosis contributes to embolism formation in upstream vascular beds under conditions of high shear stress, platelet activation, and systemic inflammation (16). Our group and others have strengthened the concept of NET-induced immunothrombosis by demonstrating that NET-rich thrombi exhibit increased mechanical stability and resistance to fibrinolysis in venous and arterial beds (7, 9, 22).

### 4.2 NETs and reperfusion failure: thrombolysis and thrombectomy resistance

NETs are strong determinants of resistance to thrombolysis. Their chromatin backbones and protease-decorated fibers inhibit the enzymatic degradation of fibrin, reducing the effectiveness of tissue plasminogen activator (tPA) and other fibrinolytics (7, 11). Ex vivo and *in vitro* studies indicate that concomitant administration of DNase I enhances fibrinolysis by disrupting chromatin structures, improving clot permeability and tPA penetration (27, 39). Similarly, early-stage clinical studies indicate that high levels of H3Cit-DNA in the circulation or thrombus are associated with poor recanalization and worse outcomes after thrombolysis. During mechanical thrombectomy, NET-rich thrombi exhibit increased structural complexity, decreased elasticity, and stronger adhesion to vessel walls, complicating complete removal and increasing the risk of distal embolization (8, 40, 41). These findings support the concept of NET burden as a biomarker for endovascular challenge and a potential therapeutic target.

### 4.3 Microvascular obstruction and the no-reflow phenomenon

Even after successful recanalization of the proximal occlusion, microvascular perfusion often remains critically impaired; this phenomenon is termed no-reflow and is strongly associated with early neurological deterioration and larger infarct volumes (42). NETs are central contributors to this process. In mouse models of middle cerebral artery occlusion, NETs accumulate densely in penumbral microvessels 3–6 h after reperfusion (Schuhmann e et al., 2021). Intravascular NETs physically occlude capillaries, trap red blood cells and platelets, promote endothelial apoptosis, activate complement (C3a, C5a), and upregulate endothelial adhesion molecules such as ICAM-1 and VCAM-1. Administration of DNase I or PAD4 inhibitors before or during reperfusion reduces microvascular obstruction, improves collateral flow, and limits infarct growth, highlighting that NETs are key drivers of microcirculatory collapse (4, 7).

### 4.4 Blood-brain barrier disruption and neurovascular damage

NETs have profound effects on the BBB. NET-derived histones, DNA, and proteases (Section 2.2) are directly cytotoxic to endothelial cells and pericytes, and MMP-9-dependent degradation of claudin-5 and occludin promotes tight junction disruption through oxidative stress and caspase-mediated apoptosis (11, 53). These changes increase the migration of neutrophils and monocytes into the ischemic parenchyma, fueling a vicious cycle of inflammation and edema. *In vivo* imaging after ischemia shows that NET-rich microvascular segments colocalize with sites of plasma leakage and hemorrhagic transformation, linking NET accumulation to BBB failure (16, 53).

### 4.5 NETs and hemorrhagic transformation after thrombolysis

Hemorrhagic transformation remains a significant complication of intravenous tPA and is a major determinant of poor outcome. NETs potentiate hemorrhagic transformation through several converging mechanisms. By weakening endothelial junctions, increasing MMP-9 and elastase activity, and promoting oxidative endothelial damage, NETs make the microvasculature more susceptible to tPA-associated bleeding. They also facilitate leukocyte extravasation into the fragile peri-infarct tissue, further destabilizing the neurovascular unit (5, 11, 48). Clinical cohorts show that patients with elevated plasma MPO-DNA or H3Cit-DNA have higher rates of hemorrhagic transformation after thrombolysis, independent of age, baseline NIHSS score, or treatment delay (39). In experimental stroke, Dnase I co-treatment attenuates HT and improves neurological outcomes, suggesting that NET-directed therapies may expand the safety window of reperfusion.

### 4.6 NET persistence: impact on neurovascular repair and clinical outcomes

Beyond the acute injury phase, persistent NETs interfere with repair and regeneration mechanisms. NET-derived histones and MPO suppress endothelial proliferation, impair vascular sprouting, and inhibit tube formation, while NE-mediated degradation of extracellular matrix components impairs perivascular remodeling (11). In the subacute and chronic stages after ischemia, NETs also maintain astrocyte activation and proinflammatory microglial states, thereby impeding synaptic plasticity and neurovascular recovery (4). Experimental reduction of NETosis enhances angiogenic signaling, increases microvascular density in peri-infarct regions, and improves long-term functional outcomes, supporting the view that NETs are negative regulators of neurovascular repair (35, 36).

Recent clinical studies further support the translational importance of NET persistency. Higher plasma or thrombus H3Cit-DNA and MPO-DNA levels are associated with larger infarct volume, early neurological deterioration, and poor functional outcome (16). NET burden in resected thrombi is independently associated with lower rates of successful recanalization after thrombolysis or thrombectomy and an increased need for multiple device passes (7, 8). Persistent NET signatures in the subacute phase have been associated with worse cognitive and neuropsychiatric sequelae, suggesting a role in long-term inflammatory remodeling (4, 43). Collectively, these data position NETs as both mechanistic contributors and clinically relevant biomarkers of ischemic stroke severity, treatment resistance, and outcomes.

## 5 NETs in hemorrhagic stroke

Neutrophil extracellular traps (NETs) are increasingly recognized as critical contributors to the pathobiology of hemorrhagic stroke, including intracerebral hemorrhage (ICH) and subarachnoid hemorrhage (SAH). Hemorrhage poses unique inflammatory and oxidative challenges, including hemoglobin, heme, iron, and thrombin, which can trigger profound neutrophil activation and NETosis in the hematoma and surrounding tissues. NETs formed in this context exacerbate perihematomal edema, BBB ​​disruption, neuronal death, and delayed cerebral ischemia, while also affecting clot stability and hematoma expansion (37–39). The following subsections summarize the mechanistic and clinical evidence for the multifaceted role of NETs in ICH and SAH.

### 5.1 NET formation in response to hemorrhagic injury

Hemorrhage rapidly activates neutrophils through numerous DAMPs released from lysed red blood cells, including hemoglobin, heme, and iron. These stimuli trigger oxidative stress and pattern recognition receptor signaling, which accelerate NETosis. Thrombin generated within the hematoma induces platelet activation and platelet-neutrophil aggregation, which are the primary drivers of vital NET release (8, 25). In parallel, damaged endothelium, astrocytes, and microglia release cytokines (IL-1β, IL-6, TNF-α), chemokines (CXCL1/CXCL2), and extracellular DNA; which further stimulates neutrophil recruitment and NET formation (33, 34).

### 5.2 NETs in intracerebral hemorrhage

#### 5.2.1 Hematoma expansion and clot stability

ICH is characterized by early hematoma expansion, which is a significant predictor of mortality. NETs accumulate within the clot and perihematomal vasculature, increasing thrombin generation, activating factor XII, and binding to tissue factor, thereby stabilizing the hematoma and promoting spreading (1, 39). NETs can also increase clot density and viscosity by trapping red blood cells.

Clinical studies have identified elevated plasma H3Cit-DNA levels in patients with early hematoma expansion, suggesting potential utility as a biomarker for risk of progression (16, 19).

#### 5.2.2 Blood-brain barrier disruption and perihematomal edema

NETs directly contribute to BBB disruption, a hallmark of perihematomal edema. Histones and NE exert cytotoxic effects on endothelial tight junction proteins (claudin-5, occludin), while MMP-9 and MPO degrade extracellular matrix components (23, 44). NETs also induce endothelial activation through TLR4 and TLR9 signaling, increasing permeability to plasma proteins such as albumin and fibrinogen.

Preclinical studies indicate that DNAse I administration or PAD4 inhibition reduces BBB leakage, alleviates vasogenic edema, and improves neurological outcomes after intracranial hemorrhage (ICH) (12, 21).

#### 5.2.3 Microglial activation and neuroinflammation

NETs act as potent activators of microglia in ICH and contribute to persistent neuroinflammation in the perihematomal tissue. Unlike ischemic stroke, the hemorrhagic environment introduces additional danger signals, particularly thrombin, hemoglobin degradation products, heme, and iron; these signals intensify and prolong microglial/macrophage activation and oxidative stress. This prolonged activation is closely associated with the development of perihematomal edema, secondary neuronal damage, and white matter damage, and may delay the transition to reparative microglial phenotypes that support hematoma resolution (12, 45).

At the mechanistic level, NET-derived DNA, histones, and proteases provide damage-associated molecular patterns detected by microglial pattern recognition and inflammation pathways, triggering cytokine release and, in some contexts, pyroptotic responses (see Sections 6-7) (11, 13). Activated microglia then enhance local inflammation through IL-1β, IL-6, TNF-α, and ROS production, potentiating leukocyte recruitment and maintaining the NET-microglia feedforward loop in perihematoma zones (12, 45).

#### 5.2.4 Effects on hematoma clearance

Although neutrophils contribute to initial erythrophagocytosis, NETosis can impair long-term clearance of blood products. Dense NET networks physically block microglia and macrophages from accessing erythrocytes, while extracellular histones inhibit phagocytic signaling (46). Therefore, NETs may inhibit hematoma uptake, promoting persistent inflammation and iron accumulation. Conceptually, limiting NET accumulation around the hematoma margin may reduce perihematomal edema and facilitate hematoma resolution, a strategy we recently discussed in detail as a potential treatment for hemorrhagic stroke (15).

### 5.3 NETs in subarachnoid hemorrhage

SAH triggers a biphasic response that includes early brain injury within 72 hours and delayed cerebral ischemia (DCI) that occurs several days later. Neutrophil recruitment to the subarachnoid space and cerebral vasculature is a hallmark of both phases, and NETs have recently been shown to be associated with vasospasm, microvascular dysfunction, and DCI.

#### 5.3.1 Vasospasm and microvascular dysfunction

NETs accumulate on the luminal surface of cerebral arteries and within the cerebral microvasculature following SAH, causing endothelial dysfunction, impairing nitric oxide signaling, and promoting vascular smooth muscle hypercontractility (11). NET-associated histones and proteases can promote vasoconstrictive responses and are increasingly playing a role in angiographic and symptomatic vasospasm. At the microvascular level, NET-rich can occlude capillaries, trap platelets and erythrocytes, and enhance local inflammatory and thrombotic signaling, thereby worsening tissue hypoperfusion in the early stages of brain injury and predisposing to DCI (47). These observations position NET accumulation as a mechanistic link between post-SAH inflammation and downstream perfusion failure.

#### 5.3.2 Neuroinflammation and delayed cerebral ischemia

SAH induces a potent neuroinflammatory response contributing to early brain damage and, particularly, to DCI in the days following the initial hemorrhage. NETosis in the subarachnoid space and along cerebral vessels enhances meningeal and perivascular inflammation, promoting endothelial dysfunction, microvascular disruption, and secondary neuronal damage that may precede or accompany DCI (11). Compared to ischemic stroke, SAH is characterized by prolonged exposure to blood-derived danger signals and persistent sterile inflammation, which can prolong the temporal window during which NET-derived inflammatory amplification remains clinically significant.

At the mechanistic level, NET-derived DNA, histones, and proteases function as damage-associated molecular patterns that activate microglia and other innate immune cells via pattern recognition and inflammation pathways (39). These shared sensing mechanisms are summarized in Sections 6-7, here we emphasize their SAH-specific link to DCI. Downstream inflammatory outputs, including cytokine and oxidative signaling, contribute to microvascular dysfunction and secondary tissue damage and may reinforce the NET-microglia feedforward loop during the high-risk period for DCI. Clinically, elevated plasma or CSF NET biomarkers have been associated with vasospasm, DCI, and poor 90-day outcome, supporting the translational importance of this pathway in SAH (16).

#### 5.3.3 NETs and meningeal inflammation

In the subarachnoid space, NETs interact with blood-derived factors and inflammatory mediators, contributing to a sustained meningeal inflammation following SAH. NET structures can support complement and coagulation components and co-localize with extracellular hemoglobin and fibrinogen, thereby concentrating danger signals at the meningeal-vascular interface and promoting sustained leukocyte recruitment. This inflammatory environment can disrupt cerebrospinal fluid dynamics and glymphatic clearance, exacerbating intracranial pressure fluctuations and potentially increasing secondary injury pathways in severe SAH (12).

### 5.4 Therapeutic modulation of NETs in hemorrhagic stroke

Given their central role in the pathophysiology of ICH and SAH, targeting NETs is a promising treatment option. Preclinical models suggest that:

• DNAse I reduces BBB leakage, edema, vasospasm severity, and neuronal apoptosis.
• PAD4 inhibitors reduce hematoma expansion and CNS inflammation.
• NE/MPO inhibitors attenuate endothelial and astrocytic damage.
• Histone neutralizing agents attenuate vasospasm and microvascular insufficiency (6, 11, 21)

Although NET-targeted interventions have not yet entered phase III trials for hemorrhagic stroke, early translational data suggest that NET inhibition may improve outcomes when combined with surgical or medical hematoma management.

**Figure 2:**
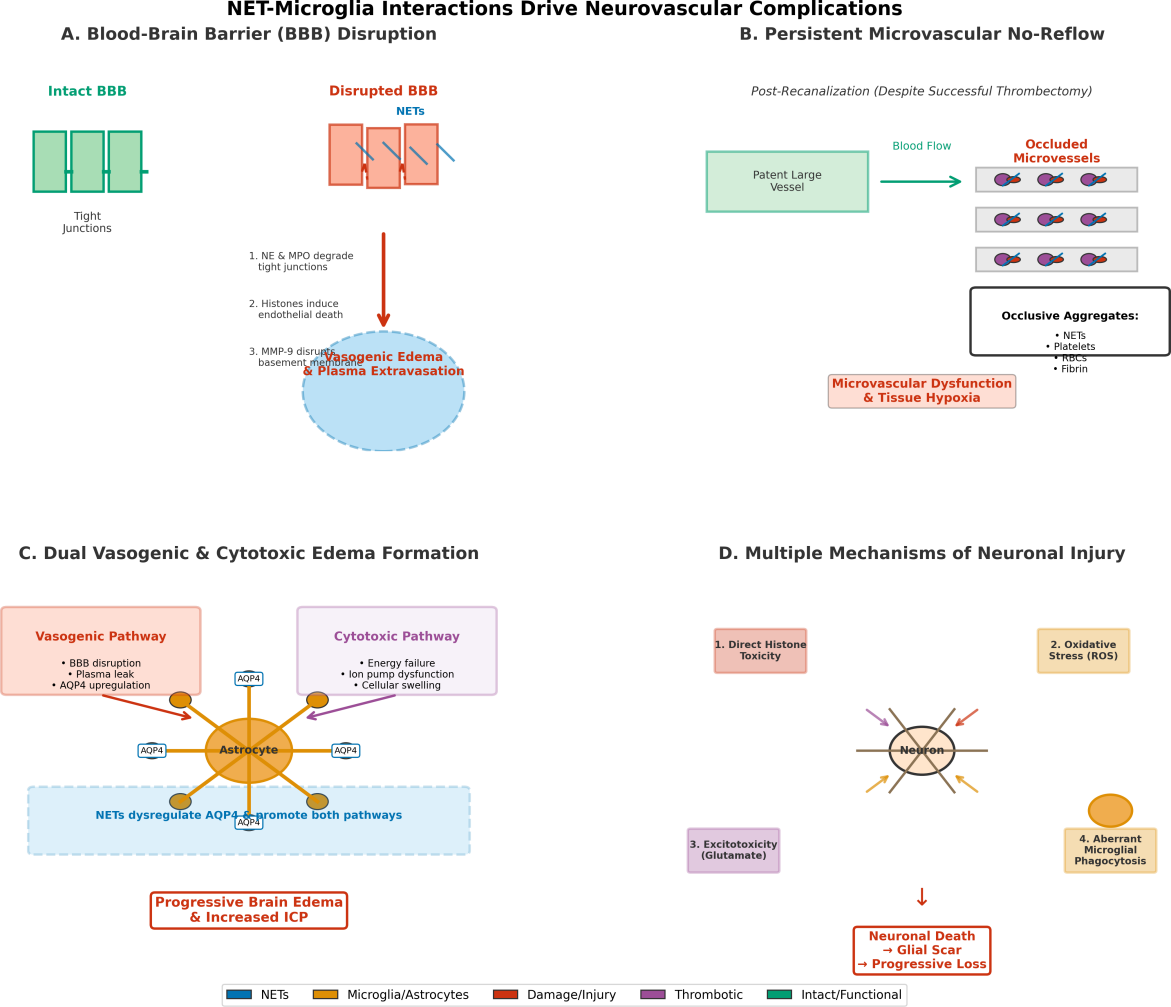
Temporal neurovascular consequences of NET-microglia interaction after stroke. NET deposition and microglial activation lead to early disruption of the blood-brain barrier and microvascular occlusion; this is followed by sustained inflammatory signaling that supports edema formation, neuronal damage, and impaired neurovascular repair in later stages. **(A)** Blood-Brain Barrier (BBB) Disruption, **(B)** Persistent Microvascular No-Reflow, **(C)** Dual Vasogenic and Cytotoxic Edema Formation, **(D)** Multiple Mechanisms of Neuronal Injury.

How these hemorrhage-induced NETs affect microglial and macrophage signaling pathways is reviewed in Sections 6 and 7. **Figure 2** illustrates the progressive neurovascular pathology cascade driven by NET-microglia interactions and illustrates (A) mechanisms of BBB disruption leading to vasogenic edema via NET-derived proteases and histones, (B) persistent microvascular reflux from NET-platelet-erythrocyte aggregates despite successful recanalization, (C) dual vasogenic and cytotoxic edema formation pathways involving AQP4 dysregulation, and (D) multiple mechanisms of neuronal injury including direct histone toxicity, oxidative stress, excitotoxicity, and aberrant microglial phagocytosis resulting in glial scar formation and progressive neuronal loss.

## 6 Bidirectional interaction between NETs and microglia/macrophages

A growing body of evidence suggests that neutrophil extracellular traps (NETs) and microglia/macrophages engage in a complex bidirectional communication that amplifies brain inflammation, accelerates tissue damage, and impairs recovery after ischemic and hemorrhagic stroke. This interaction is mediated by pattern recognition receptors, inflammasome platforms, chemokine networks, cytokine loops, and extracellular proteases, creating a self-reinforcing inflammatory loop within the neurovascular unit (30). In this section, we focus on the interaction between NETs and microglia/macrophages at the cellular level.

The following sections summarize the mechanistic basis of this immune interaction.

### 6.1 Microglial sensing of NET components

NET components act as potent DAMPs that directly stimulate microglial receptors. These interactions activate inflammatory signaling cascades that drive cytokine production, inflammasome formation, and pyroptotic cell death. Because these sensing pathways are shared to stroke subtypes, we summarize them here as a unified mechanistic framework before returning to ischemic and hemorrhagic contexts.

#### 6.1.1 TLR9 signaling

NET-derived double-stranded DNA (dsDNA) is a potent agonist of microglial TLR9 and leads to MyD88-dependent activation of the NF-κB and type I interferon pathways. This signaling leads to release of IL-1β, IL-6, and TNF-α, upregulation of CCL2/CCR2, increasing monocyte recruitment, increased expression of ICAM-1 and VCAM-1 in endothelium and enhanced stimulation of circulating neutrophils. These effects are evident in peri-infarct regions and perihematomal tissue following intracranial hemorrhage (ICH) (13, 38, 48).

#### 6.1.2 cGAS-STING activation

Cytosolic leakage of NET DNA into microglia activates cGAS, generating cGAMP and activating STING, with downstream induction of interferon-stimulated genes (ISGs). Results include robust IFN-β release, sustained microglial activation in subacute phases, and persistence of inflammation. cGAS-STING activation is particularly important in SAH, where NET D is abundant in the subarachnoid space (39).

#### 6.1.3 NLRP3 inflammasome engagement

NET histones and oxidized mitochondrial DNA trigger NLRP3 inflammasome activation in microglia and macrophages (11). This leads to caspase-1 activation, IL-1β and IL-18 release, pyroptotic cell death, and increased neuroinflammation. NLRP3-mediated cytokine release contributes to BBB breakdown and microvascular dysfunction in IS and increases perihematomal edema in ICH (12).

#### 6.1.4 AIM2 inflammasome activation by NET DNA

AIM2 recognizes cytosolic dsDNA from NETs, forming inflammasome complexes that activate caspase-1 independently of NLRP3. AIM2 activation plays a role in microglial pyroptosis, white matter damage, and prolonged inflammatory signaling in chronic phases. This pathway is increasingly recognized in both ischemia and SAH (45).

#### 6.1.5 Histone-mediated cytotoxicity and microglial activation

NET-derived histones (especially H3 and H4) exert potent cytotoxic and proinflammatory effects to disrupt microglial membranes, stimulate TLR2 and TLR4 signaling, and trigger ROS production and mitochondrial dysfunction (5, 11).

These pattern recognition and inflammasome pathways are described in more detail in Section 7.

### 6.2 Microglia/macrophage-derived amplification of NETosis and feedforward signaling cycles

Activated microglia and macrophages can promote NET formation and maintain neutrophil recruitment, thereby closing the feedback loop in the NET-microglia axis. Pro-inflammatory mediators released from activated microglia/macrophages, including IL-1β and TNF-α (and related cytokine/chemokine programs), predispose circulating neutrophils to increased activation, ROS production, and NETosis, while simultaneously enhancing endothelial activation and leukocyte adhesion within peri-infarct and perihematomal microvessels. Among these signals, IL-1β is frequently shown to be a potent enhancer of chromatin remodeling pathways associated with NETosis in neutrophils (38, 48).

In addition to soluble cytokines, purinergic and oxidative pathways provide strong amplification signals. Microglial ATP release activates neutrophil P2X7 receptors, leading to Ca²^+^ influx, mitochondrial ROS generation and NLRP3-mediated NETosis. This mechanism has been demonstrated in models of ischemia and hemorrhage (14). Microglia/macrophage-derived ROS and NET-associated oxidant activity further potentiates oxidative stress within the neurovascular unit, which may facilitate ongoing neutrophil activation and NET release (11).

These cellular interactions are stabilized by broader chemokine-endothelium-protease cycles that support inflammatory cell trafficking and blood-brain barrier (BBB) ​​sensitivity. Microglial/macrophage enhance chemokine signaling (e.g., CXCL1/CXCL2 and CCL2-linked recruitment programs) that promotes the continued influx of neutrophils and monocytes into regions enriched with NET material, while endothelial activation increases adhesion molecule expression and promotes leukocyte recruitment in damaged microvessels (11, 48). Proteases such as MMP-9 further destabilize the BBB by cleaving tight junction proteins and extracellular matrix components. This facilitates additional leukocyte extravasation and increasing the exposure of perivascular microglia to circulating DAMPs (11). HMGB1, which can bind to NET structures, and functions as an additional potent amplifier of microglial activation and leukocyte recruitment in these inflammatory niches (48, 49). Together, these cytokine-, purinergic-, oxidative-, and endothelium-mediated cycles explain how microglia/macrophages not only respond to NET-induced danger signals but also actively maintain NETosis and immunothrombosis, prolonging post-stroke neurovascular dysfunction and inflammatory damage.

### 6.3 Spatial and temporal dynamics of crosstalk

Spatial transcriptomic and immunofluorescence studies indicate that NET-microglia interactions are not uniformly distributed but concentrated in distinct microdomains. In the peri-infarct microvasculature, dense capillary NET deposits co-localize with clusters of activated microglia, creating microthrombotic “hot spots” that impede reperfusion. Along the edges of ICH hematomas, extensive NET sheets lie adjacent to reactive microglia and infiltrating macrophages, providing a structural scaffold for ongoing immunothrombosis. In subarachnoid hemorrhage, NETs accumulate along meningeal vessels in the subarachnoid space and trigger microglial activation in overlying cortical regions (12, 49). These spatially restricted niches likely represent critical targets for localized therapeutic interventions.

Temporally, NET-microglia crosstalk unfolds in several overlapping stages. During the hyperacute phase (0–6 h), rapid neutrophil infiltration and early NETosis coincide with microglial transition to a surveillance phenotype. In the acute phase (6–72 h), NET accumulation peaks and is accompanied by robust activation of microglial NLRP3 inflammasomes and cGAS-STING signaling. In the subacute phase (days 3–14), incompletely cleared NET fragments and persistent DAMPs delay the shift of microglia toward a reparative phenotype. In the chronic phase (weeks), residual NET material and ongoing low-grade inflammation contribute to sustained microglial activation and smoldering neurodegeneration (4, 50).

### 6.4 Extracellular vesicles in NET-microglia communication

Extracellular vesicles (EVs), including exosomes and microvesicles, add another layer of complexity to NET-microglia communication. Neutrophil-derived EVs can carry oxidized DNA, granule proteins, and MPO, which activate microglial STING signaling and sustain type I interferon responses (38). Conversely, microglial EVs carry proinflammatory microRNAs such as IL-1β, miR-155, and enzymes that regulate redox balance, thereby predisposing neutrophils for enhanced NETosis (51). Phosphatidylserine-enriched EVs also facilitate physical interactions between neutrophils and microglia within inflammatory microvascular niches. This emerging field suggests that EVs may represent both biomarkers and potential delivery vehicles for NET- or microglia-modulating therapies.

## 7 Molecular mechanisms of crosstalk

Interactions between neutrophil extracellular traps (NETs) and microglia/macrophages are orchestrated by a complex molecular network involving pattern recognition receptors, cytokines, chemokines, proteases, DAMPs, and bioactive lipids. Collectively, these pathways trigger immunothrombosis, promote neurovascular damage, and determine the severity of both ischemic and hemorrhagic stroke. Understanding these molecular mechanisms is important for identifying applicable therapeutic targets.

### 7.1 HMGB1–platelet–NET axis

High-mobility group box protein 1 (HMGB1) is a potent nuclear DAMP released by necrotic neurons, activated microglia, and platelets in stroke. It acts as a central initiator of NET-microglia interactions through several complementary effects. First, HMGB1 directly primes neutrophils for NETosis. TLR4 and RAGE interaction on neutrophils activates PAD4, triggering histone citrullination, chromatin decondensation, and NET release (26, 49). Platelets exposed to HMGB1 form platelet-neutrophil clusters that localize to cerebral microvessels and further aggravate vital NETosis, thereby increasing the NET burden in the ischemic and perihematomal microcirculations (8). Second, NETs enhance microglial activation by binding to and stabilizing HMGB1. NET DNA and histones associate with HMGB1 with high affinity to form HMGB1-NET complexes that persist in the extracellular space. These complexes potently stimulate microglial TLR4, RAGE, and TLR2 signaling, leading to the potent production of IL-1β, TNF-α, and IL-6, the upregulation of chemokines such as CXCL1/2 and CCL2, increased oxidative and mitochondrial stress, and secondary endothelial activation (26, 52). Third, HMGB1 promotes microvascular thrombosis and immunothrombosis. By enhancing platelet activation, tissue factor expression, and fibrin deposition, HMGB1 synergizes with NET scaffolds to stabilize intravascular thrombi and propagate immunothrombosis in peri-infarct and perihematomal regions (52). Together, these mechanisms position the HMGB1-platelet-NET axis as a key driver of NET-microglia amplification and a promising therapeutic target in both ischemic and hemorrhagic stroke.

### 7.2 TLR4 and TLR9 signaling in NET-induced microglial activation

Building on the overview in Section 6.1, PRRs on microglia, particularly TLR4 and TLR9, recruit NET components to intracellular signaling cascades. In stroke models, TLR4 activation by NET histones and HMGB1 contributes to BBB disruption in ischemic stroke and vasospasm after SAH, while TLR9-mediated NET DNA sensing induces sustained inflammatory responses and chemokine upregulation. Preclinical TLR9 inhibition reduces infarct size and perihematomal inflammation, highlighting these DNA sensing pathways as attractive therapeutic targets (11, 46, 49, 50).

### 7.3 MMP-mediated BBB disruption

NET-derived enzymes (NE, MPO) and microglia/macrophage-derived matrix metalloproteinases (specifically MMP-3 and MMP-9) synergize to disrupt the integrity of the BBB. The mechanisms include degradation of tight junction proteins (occludin, claudin-5), degradation of the basal lamina, endothelial apoptosis via proteolytic stress, and increased leukocyte migration (11, 53). This protease-induced BBB breakdown significantly contributes to edema, hemorrhagic transformation, and neuronal death.

### 7.4 Complement interactions with NETs and microglia

NETs activate complement through classical, lectin, and alternative pathways. C3a and C5a stimulate microglial inflammatory activation, attract neutrophils, and potentiate NETosis in a feedforward manner. C5b-9 (MAC) damages the endothelium and worsens microvascular obstruction by promoting NET formation (47). In SAH, complement-NET interactions significantly contribute to vasospasm.

### 7.5 Oxidative and mitochondrial pathways

Microglial ROS generated by NADPH oxidase (NOX2) and dysfunctional mitochondria serve as upstream drivers of NETosis (48). Conversely, NET-derived MPO and histones impair mitochondrial function in microglia, increase mitochondrial ROS (mtROS), and trigger the cGAS–STING and inflammasome pathways. This reciprocal oxidative damage exacerbates inflammatory damage.

### 7.6 Integrative view: a self-perpetuating immunothrombotic cycle

Taken together, the available evidence supports a model in which NETs and microglia/macrophages form a self-sustaining immunothrombotic circuit in both ischemic and hemorrhagic stroke. Ischemia, mechanical vascular injury, or exposure to heme, iron, and thrombin rapidly activate platelets and neutrophils, leading to the formation of platelet-neutrophil aggregates in large artery thrombi and downstream microvessels and PAD4-dependent NET release (7). NET scaffolds capture erythrocytes and platelets, concentrate tissue factor and HMGB1, and stabilize intravascular thrombi, promoting microvascular non-reflow and providing a highly immunogenic reservoir for DAMPs (42).

In parallel, NET-derived components are sensed by microglia via pattern recognition receptors and inflammasome platforms (Section 6.1), directing NF-κB and type I interferon signaling and activating NLRP3 and AIM2 inflammasomes (11, 45). Activated microglia and infiltrating macrophages secrete IL-1β, IL-18, TNF-α, IL-6, CXCL1/2, and CCL2, increase MMP-9 and ROS production, and disrupt the BBB, thereby enhancing leukocyte recruitment and facilitating further NET accumulation in the injured microvasculature (48, 49). Overlapping temporal waves of NET formation and microglial activation (Section 6.4) delay the transition to reparative phenotypes and prolong tissue fragility.

Over time, this NET-microglia axis links intravascular thrombosis to parenchymal neuroinflammation: Immunothrombosis in the macro- and microcirculation promotes blood-brain barrier (BBB) breakdown, vasogenic edema, and secondary ischemic damage, while persistent inflammatory microglia and macrophages inhibit angiogenesis, impair synaptic plasticity, and contribute to chronic neurodegeneration (48–51). Conceptually, this integrative perspective helps explain why interventions targeting a single node (e.g., NET depletion or microglial suppression alone) have shown only partial benefit in preclinical studies. Effective immunomodulation in stroke will likely require time-adapted strategies that reduce NET burden and reprogram microglial/macrophage responses, while preserving essential host defense and repair functions (6, 7).

**Figure 3:**
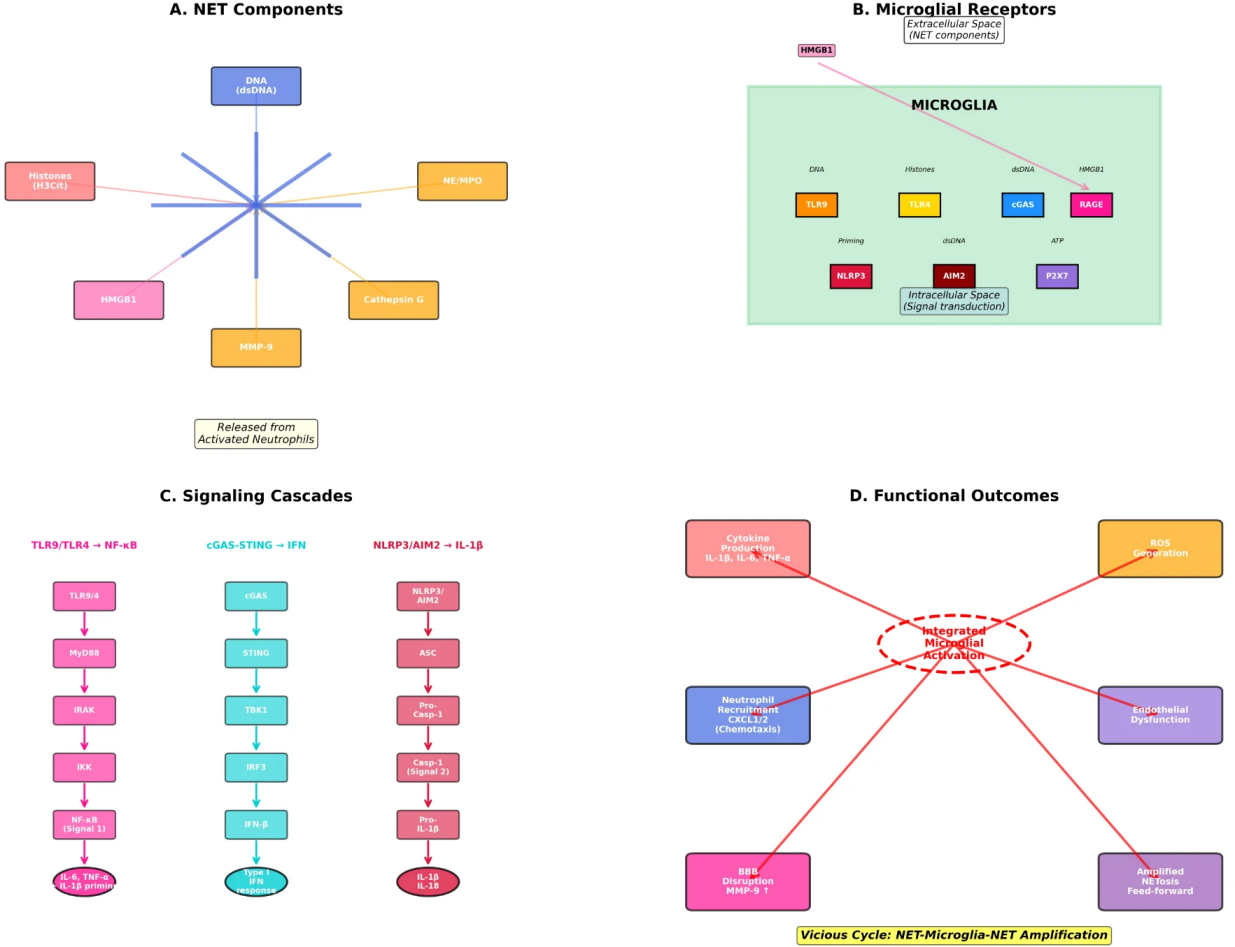
Progressive neurovascular pathology driven by NET-microglia interactions after stroke. NET-derived components (DNA, histones, proteases, and HMGB1) activate microglial pattern recognition and inflammation pathways, promoting cytokine release, oxidative stress, and leukocyte recruitment. Mutual signaling from activated microglia and macrophages sustains NET formation and immunothrombosis within the neurovascular unit. These feedforward interactions contribute to disruption of the blood-brain barrier, microvascular dysfunction, edema formation, and secondary neuronal damage throughout ischemic and hemorrhagic stroke. **(A)** NET Components, **(B)** Microglial Receptors, **(C)** Signaling Cascades, **(D)** Functional Outcomes.

The major molecular pathways regulating NET-microglia crosstalk are systematically summarized in **Table 1**, which details specific ligands, receptors, downstream signaling cascades and their functional consequences in the context of stroke pathophysiology. **Figure 3** details the molecular architecture of NET-microglia communication, detailing (A) specific NET-derived components (DNA, histones, proteases, HMGB1) that serve as damage signals, (B) their interactions with microglial pattern recognition receptors including TLR4, TLR9, cGAS, RAGE, and the inflammasome sensors NLRP3 and AIM2, (C) downstream signaling cascades through NF-κB, STING, and caspase-1 activation pathways, and (D) integrated functional consequences including cytokine storm, ROS production, neutrophil recruitment, and BBB disruption, which collectively constitute the vicious cycle of NET-microglia-NET amplification.

**Table 1:** Key molecular pathways linking NETs and microglia/macrophages in stroke.

Pathway/ligand	Source	Receptor/target in microglia	Downstream effects	Stroke context
NET-derived dsDNA	Neutrophils (NETs)	TLR9, cGAS–STING	NF-κB activation, type I IFN responses, IL-1β release, microglial priming	IS, ICH, SAH
Histones (H3/H4)	NET cores	TLR2/TLR4	Membrane damage, inflammasome priming, ROS generation	IS, ICH
HMGB1–NET complexes	Damaged neurons, NETs	RAGE, TLR4	Amplified cytokine production, endothelial activation, microthrombosis	IS, SAH
NE/MPO	Neutrophil granules	— (protease-dependent)	BBB disruption, ECM disruption, microvascular damage	IS, ICH
Microglial IL-1β/IL-18	Activated microglia	Neutrophil priming via systemic circulation	Enhanced neutrophil recruitment, ↑ NET formation, ↑ PAD4 activity	IS, ICH, SAH
TNF-α, IL-6	Microglia/macrophages	Endothelial ICAM-1/VCAM-1 upregulation	Increased leukocyte adhesion and transmigration, vascular inflammation	IS, ICH
CXCL1/CXCL2	Microglia/macrophages	Neutrophil CXCR2	Chemotactic neutrophil recruitment to injury sites	IS, ICH, SAH
ATP → P2X7	Damaged cells, microglia	Neutrophil P2X7	Ca²^+^ influx, mitochondrial ROS, NLRP3-dependent NETosis	IS, ICH
MMP-9	Microglia/macrophages	ECM substrates	Tight junction disruption, BBB disruption, NET extravasation	IS, ICH
Complement (C5a, MAC)	Plasma, NET activation	C5aR (microglia)	Microglial activation, NET amplification	SAH

## 8 Therapeutic targeting strategies

Given the central role of neutrophil extracellular traps (NETs) and microglia/macrophages in the propagation of thrombo-inflammatory response, BBB disruption, and neuronal injury, therapeutic targeting of this axis is gaining increasing attention. Interventions aimed at inhibiting NET formation, promoting NET degradation, or regulating microglial activation have shown promising results in preclinical models such as ischemic stroke (IS), intracerebral hemorrhage (ICH), and subarachnoid hemorrhage (SAH) (4, 12). Combination approaches that simultaneously target neutrophil and microglial signaling offer a particularly promising strategy for interrupting the previously described vicious cycle immunothrombotic cycle. Based on this mechanistic framework, several groups have proposed co-targeting neutrophils and microglia as a rational immunotherapeutic strategy in ischemic stroke (6, 16, 23, 31, 47).

### 8.1 Inhibition of NET formation

The first strategy is to prevent NET formation at its source. PAD4 inhibitors such as GSK484 and BB-Cl-amidine have become prototypical NET blockers, targeting histone citrullinated step. In experimental ischemic stroke, prophylactic or early post-occlusion administration of PAD4 inhibitors reduces NET burden in large artery thrombi and microvessels, limits infarct size, and improves neurological outcomes without completely abolishing neutrophil recruitment (6, 7). Similar benefits have been reported in preclinical ICH models, where PAD4 inhibition reduces perihematomal edema and improves hematoma resolution (15).

Upstream signaling pathways offer additional intervention points. Purinergic P2X7 signaling drives Ca²^+^ influx, mitochondrial ROS, and NLRP3-dependent NETosis; selective P2X7 antagonists and NOX2 inhibitors reduce NET formation and BBB damage in experimental stroke but have not yet entered clinical trials (14). Blocking neutrophil recruitment via CXCR2 antagonists or targeting platelet-neutrophil interactions (e.g., via P-selectin or GPIIb/IIIa inhibitors) has also been shown to reduce intravascular NET accumulation and microvascular no-reflow, although concerns remain about infection risk and interference with hemostasis (8, 42). Collectively, these approaches aim to blunt the initial wave of NET formation that initiates the NET-microglia amplification cycle.

### 8.2 Degrading NETs and neutralizing NET components

A second important strategy is to disintegrate existing NETs and neutralize their toxic payload. Recombinant DNase I, currently used clinically for cystic fibrosis, effectively degrades extracellular DNA structures, and reduces NET density in ischemic cerebral microvessels. In rodent stroke models, DNase I enhances tPA-mediated thrombolysis, improves microvascular reperfusion, reduces BBB leakage, and attenuates hemorrhagic transformation (7, 39). DNase1L3, which primarily targets nucleosome-bound chromatin, may offer complementary activity against more compact NET structures, but its role in stroke has not yet been investigated. Our group and others have proposed DNase-based approaches as promising adjuncts in hemorrhagic stroke, particularly for reducing perihematomal edema and promoting hematoma clearance (15, 49–53).

Similarly, strategies that neutralize NET-associated histones and proteases aim to reduce downstream toxicity. Non-anticoagulant heparin derivatives activated protein C, and recombinant thrombomodulin can bind to extracellular histones, reducing endothelial damage and vasospasm in experimental SAH and IS (54). NE inhibitors (such as alvelestat) and MPO can protect BBB integrity and reduce infarct volume by limiting protease-mediated tight junction degradation and oxidative stress (47). Neonatal NET inhibitory factor (nNIF) and related peptides represent an innovative class of endogenous NET modulators; in preclinical models of sepsis and ischemia, these peptides reduce NET burden and improve survival without significant immunosuppression (55, 56). Combined, these approaches transform NETs from rigid, prothrombotic structures into more manageable remnants and blunt their most detrimental functions.

### 8.3 Modulation of microglial and macrophage responses

Because microglia and infiltrating macrophages play a central role in the propagation of NET-induced inflammation, a complementary strategy is to reprogram these cells rather than suppress them overall. NLRP3 inflammasome inhibitors, such as MCC950 and the orally active agent dapansutril, reduce IL-1β/IL-18 production, microglial pyroptosis, and BBB breakdown in experimental stroke (35). In intracranial hemorrhagic shock models, NLRP3 inhibition reduces perihematomal edema and improves functional recovery, supporting the use of inflammasome blockade as an adjunct to NET-targeted therapies (30).

Altering microglial survival and phenotype is another promising avenue. CSF1R inhibitors (e.g., PLX3397, PLX5622) transiently deplete microglia or shift them toward less inflammatory states, which reduces infarct volume and cytokine release in some studies; however, concerns remain about impaired debris clearance and long-term effects on cognition (17, 33). In contrast, TREM2 agonist antibodies promote phagocytosis, debris clearance, and an anti-inflammatory microglial profile; in hemorrhagic stroke models, TREM2 activation accelerates hematoma resolution and improves outcomes (34). Our own work has highlighted the potential of coordinated immunomodulation of microglia/macrophages and neutrophils as a rational strategy in both ischemic and hemorrhagic stroke (30, 57). Consequently, successful microglial therapies will need to preserve or enhance protective functions such as efferocytosis and trophic support while reducing harmful inflammatory outputs.

### 8.4 Combination and sequential strategies targeting the NET-microglia axis

Given the complex interaction between NETs and microglia/macrophages, monotherapy targeting a single node may not be sufficient to disrupt the self-perpetuating immunothrombotic cycle described above. Conceptually, combination and sequential approaches are attractive. One example is pairing DNase I with an NLRP3 inhibitor: the former reduces intravascular NET structures and improves microvascular flow, while the latter limits microglial and macrophage inflammasome activation triggered by residual NET fragments and DAMPs (14, 35). Another strategy is to use PAD4 inhibition or CXCR2 blockade in the hyperacute phase to limit NET formation, followed by delayed TREM2 agonism or CSF1R modulation in the subacute phase to promote reparative microglial phenotypes and promote neurovascular remodeling (14, 30).

Designing such regimens will require careful attention to timing, dosage, and infection risk, as well as rigorous biomarker and imaging strategies to identify patients with high NET burden and maladaptive microglial activation. In the following sections, we discuss how circulating NET markers, thrombus composition, microglia-focused PET imaging, and AI-assisted clinical decision support can be used to guide these NET-microglia-targeted interventions on an individualized basis. For now, preclinical data strongly suggest that multitargeted, temporally phased immunomodulation offers the greatest promise for safely attenuating thromboinflammation while preserving essential host defenses in ischemic and hemorrhagic stroke.

The full spectrum of treatment strategies targeting NET formation, NET degradation, and microglial signaling is summarized in **Table 2**, highlighting the most promising preclinical interventions and their mechanistic targets.

**Table 2:** Therapeutic strategies targeting the NET-microglia axis in stroke treatment.

Treatment strategy	Mechanism of action	Primary molecular targets	Preclinical evidence	Clinical situation	Key references
Net formation blockage					
PAD4 Inhibitors (GSK484, BB-Cl-amidine, Cl-amidine)	Block histone citrullination, necessary for chromatin decondensation	Peptidylarginine deiminase 4 (PAD4)	Reduction in infarct volume and improvement in outcomes in MCAO models; decreased NET burden in the thrombus	Preclinical stage only	(7, 22, 39)
Neutrophil Depletion (Anti-Ly6G antibodies)	Reduce cellular NET supply	Ly6G+ neutrophils	Reduction in infarct size and BBB impairment in rodent IS models; reduced hemorrhagic transformation	Preclinical stage only (safety concerns)	(8, 27)
P2X7 Receptor Antagonists (A438079, Brillante)	Block ATP-induced NET release	P2X7 purinergic receptor	Reduction in NETosis in IS models; decreased neuroinflammation	Preclinical stage; Brillante in early clinical development phase	(40, 41)
NOX2 Inhibitors (Apocynin, GSK2795039)	Reduce ROS-induced NETosis.	NADPH oxidase 2 (NOX2)	Reduced oxidative stress and NET formation in IS and ICH models	Preclinical stage only	(16, 42)
Net degredation strategies					
Recombinant DNase I (Pulmozyme/Dornase alfa)	Cleaves DNA backbone of NETs, increases thrombolysis	Extracellular DNA	Improved recanalization and reduced infarction in animal models; increased tPA efficacy	Phase I/II clinical trials in acute ISIS (proven safety)	(7, 11, 27)
DNase 1L3 Supplementation	Increases endogenous NET clearance capacity	Extracellular chromatin	Improved NET degradation in hemorrhagic stroke models	Preclinical studies only	(43)
Histone Neutralizers (Heparin, Active Protein C)	Binds to and neutralizes cytotoxic extracellular histones	Histones H2A, H2B, H3, H4	Reduced endothelial damage and blood-brain barrier disruption in multiple stroke models	Heparin: clinical use (anticoagulation); APC: terminated studies	(8, 44)
Microglial/macrophage modulation					
NLRP3 Inflammation Inhibitors (MCC950, Dapansutril/OLT1177)	Block NLRP3-mediated IL-1β and IL-18 release	NLRP3, caspase-1	Reducing inflammation and improving outcomes in ischemic stroke, intracranial hemorrhage, and subarachnoid hemorrhage models	Dapansutril in Phase II studies for cardiovascular inflammation	(31, 45)
TREM2 Agonists (AL002, anti-TREM2 mAb)	Promote anti-inflammatory M2-like microglia polarization	TREM2 receptor	Enhancing debris clearance and neuroprotection in hemorrhagic stroke	AL002 in Phase I (neurodegeneration); stroke studies ongoing	(30, 46)
CSF1R Inhibitors (PLX5622, PLX3397)	Regulate microglia proliferation and phenotype	Colony-stimulating factor 1 receptor	Reducing microglia activation in ischemic models; effects vary depending on timing	Preclinical studies only	(47)
Anti-inflammatory Biologics (Anakinra, anti-TNF, anti-IL-6)	Neutralize pro-inflammatory cytokines	IL-1R, TNF-α, IL-6	Reducing inflammation in various stroke models	Anakinra: Phase II IS studies completed; anti-IL-6: ongoing studies	(48, 49)
Combination approaches					
DNase I + NLRP3 Inhibitor	Simultaneous degradation of NETs and inhibition of inflammation	Extracellular DNA + NLRP3	Synergistic neuroprotection in preclinical IS models	Preclinical stage only	(50)
PAD4 Inhibitor + TREM2 Agonist	Preventing NET formation while supporting microglia repair	PAD4 + TREM2	Enhancing functional recovery in experimental stroke	Preclinical stage only	(51)
Histone Neutralization + IL-1 Blockade	phenotype Neutralizing NET toxicity and inhibiting cytokine amplification	Histones + IL-1β pathway	Reducing blood-brain barrier disruption and edema in intracranial hemorrhage models	Preclinical stage only	(52)

## 9 Clinical translation and biomarker potential

Translating mechanistic insights into NET-microglia/macrophage interactions into clinical practice requires reliable biomarkers, advanced imaging tools, and well-designed intervention studies. Over the past decade, NET-associated molecular signatures have emerged as promising biomarkers for stroke severity, treatment response, hemorrhagic transformation, and long-term outcomes (16). Parallel advances in thrombus pathology and neurovascular imaging have increased the feasibility of personalized treatment strategies targeting NETs and microglial activation.

### 9.1 Circulating biomarkers of NET formation

Circulating NET components have emerged as promising tools for risk stratification and treatment monitoring in stroke. Complexes of citrullinated histone H3 with DNA (H3Cit-DNA) and myeloperoxidase with DNA (MPO-DNA) are the most widely studied, reflecting PAD4-dependent chromatin decondensation and neutrophil activation, respectively. Several cohorts have shown that higher plasma H3Cit-DNA or MPO-DNA levels at presentation are associated with larger infarct volume, more severe neurological deficit, and worse 90-day outcome after ischemic stroke, independent of age and vascular risk factors (16). Similar associations have been reported in ICH and SAH, where elevated NET signatures predict perihematomal edema expansion and delayed cerebral ischemia (5, 7, 58).

Other NET-associated markers provide complementary information. Cell-free DNA and nucleosomes reflect global cell death and NETosis but are less specific; HMGB1 and S100A8/A9 integrate signals from NETs, ​​microglia, and damaged neurons; and plasma neutrophil elastase or MPO levels capture protease-induced vascular damage (11, 58). In principle, longitudinal measurement of these markers could help identify patients with persistent NET activity who may benefit from extended DNase or PAD4 inhibitor therapy or flag those at high risk of hemorrhagic transformation after thrombolysis. However, pre-assay variability, lack of standardized assays, and overlap with systemic inflammatory conditions remain important limitations that need to be addressed before implementation into routine clinical practice.

### 9.2 CSF and microglia-associated biomarkers

Cerebrospinal fluid (CSF) provides a more direct window into central nervous system inflammation and microglial activation. CSF concentrations of soluble TREM2 (sTREM2), YKL-40 (CHI3L1), and neurofilament light chain reflect microglial activation, astrocytosis responses, and axonal injury, respectively. Elevated levels of sTREM2 and YKL-40 have been reported in acute ischemic stroke, ICH, and SAH; these levels are associated with infarct size, perihematomal edema, and delayed cerebral ischemia (43, 51). CSF IL-1β, IL-6, and TNF-α capture inflammasome and NF-κB activity and are often markedly increased in patients with severe SAH or malignant middle cerebral artery infarction.

NET-derived components can also be detected in CSF. H3Cit–DNA and MPO–DNA complexes have been identified in SAH and ICH, where they are enriched in patients with vasospasm and poor functional outcomes. CSF MMP-9 and S100B reflect BBB disruption and astrocyte injury and are often observed in conjunction with radiological evidence of edema and hemorrhagic transformation. Although CSF sampling is more invasive and therefore often limited to patients with ventricular drainage or neurocritical care monitoring, such biomarker panels may be invaluable for mechanistic trials of NET- or microglia-targeted therapies in severe stroke (59, 60).

### 9.3 Thrombus NET content as a tissue biomarker

Mechanical thrombectomy offers a unique opportunity to obtain intravascular tissue for histological and molecular analysis. Immunostaining of the resulting thrombi has consistently demonstrated abundant NET structures, defined by the co-localization of citrullinated histone H3, MPO, and DNA (7, 9). High NET burden in thrombi is associated with increased fibrin density, greater mechanical stiffness, and resistance to both intravenous thrombolysis and endovascular recovery, often requiring multiple device passes and resulting in lower complete reperfusion rates (8, 41). In some series, NET-rich thrombi also predict a higher risk of distal embolization and hemorrhagic transformation.

Beyond simple quantification, spatial mapping of NETs within thrombi (e.g., localization in the outer shell versus core) can provide insights into clot evolution and inform device design or adjunctive pharmacotherapy. Flow cytometry and proteomic analyses of thrombus digests can further characterize associated neutrophil, platelet, and complement signatures. In the future, thrombus NET burden could be integrated into decision algorithms for additional DNase, PAD4 inhibitors, or intensified antithrombotic strategies in select patients.

Mechanical thrombectomy has enabled the direct study of human stroke thrombi. Key findings include (7, 8, 16, 58):

• NET-rich thrombi require more thrombectomy, increasing procedure complexity.
• High NET content predicts lower ultimate reperfusion success.
• NET density correlates with clot age, suggesting that NETs accumulate as the thrombus matures.
• NETs form complex scaffolds with fibrin and platelets, explaining thrombolytic resistance.

This information supports the integration of NET biomarkers into reperfusion decision-making algorithms and encourages NET-targeted adjunctive therapies to thrombectomy.

### 9.4 *In vivo* imaging of NET-microglia activation

Non-invasive imaging of the NET-microglia axis is an active area of ​​development. Positron emission tomography (PET) tracers targeting the 18 kDa translocator protein (TSPO), such as [(11)C]PK11195 vs [(18)F]DPA-714, have been widely used as markers of microglial activation in stroke and have been associated with inflammatory burden and functional outcomes (33, 61). While TSPO is not specific for detrimental and reparative microglial states, serial TSPO-PET imaging can monitor the temporal evolution of neuroinflammation and serve as a pharmacodynamic indicator for microglia-focused therapies.

More specific probes are emerging. Myeloperoxidase-targeted MRI or PET agents enable imaging of neutrophil activity and, consequently, NET-associated inflammation in ischemic and hemorrhagic lesions (47). Dynamic contrast-enhanced MRI (DCE-MR) and CT or MR perfusion imaging capture important downstream consequences of NET-microglia crosstalk by measuring BBB permeability and microvascular flow. Experimental NET-specific PET tracers, including radiolabeled antibodies against H3Cit or DNA-histone complexes, are being investigated in preclinical models and may eventually allow direct *in vivo* quantification of NET burden (62, 63). Multimodal imaging combining microglial activation, neutrophil activity, BBB integrity, and perfusion may ultimately provide a comprehensive picture of immunothrombosis in individual patients.

### 9.5 AI-enabled biomarker and imaging integration

The growing diversity of circulating biomarkers, tissue markers, and imaging modalities raises a fundamental question: How can clinicians integrate this heterogeneous data to guide treatment in real time? Artificial intelligence (AI) offers a powerful framework for this challenge. Machine learning models can use clinical variables, structural and perfusion imaging, thrombus characteristics, and longitudinal NET/microglia biomarkers to predict outcomes such as infarct enlargement, hemorrhagic transformation, or edema expansion. In other vascular and device-related fields, we and others have shown that AI-powered decision support can improve risk prediction, optimize device selection, and navigate complex regulatory environments (64–66).

Adapting these concepts to stroke could provide models that routinely estimate NET burden and microglial activation states from existing data, identify patients most likely to benefit from DNase, PAD4 inhibition, or NLRP3 blockade, and recommend the optimal timing and duration of such treatments (67–69). In severe ICH or SAH, AI tools can integrate CSF biomarkers, neuroimaging, and physiological parameters to predict delayed cerebral ischemia or hematoma expansion and trigger early escalation of immunomodulatory therapy. Realizing this vision will require compatible multicenter datasets, transparent model reporting, robust external validation, and regulatory frameworks that ensure safety, fairness, and explainability (64, 66). However, AI-guided integration of NET and microglial biomarkers represents a logical next step toward precision immunotherapy in stroke.

To support clinical translation, **Table 3** provides an overview of established and emerging biomarkers associated with NET activity and microglial activation and presents their diagnostic and prognostic significance across stroke subtypes.

**Table 3:** Biomarker and imaging approaches for NET-microglia axis assessment.

Biomarker category	Specific biomarker	Source/sample type	Detection method	Clinical correlation	Timing	References
Blood biomarkers - net markers						
NET-DNA Complexes	H3Cit-DNA Complexes	Plasma/Serum	ELISA, immunofluorescence	Elevated in ischemic stroke, intracranial hemorrhage, and subarachnoid hemorrhage; associated with infarct volume and poor functional outcomes	Peak 6–24 hours	(7, 9, 22)
	MPO-DNA Complexes	Plasma/Serum	ELISA, capture test	Indicates NET activity; predicts hemorrhagic transformation and thrombectomy resistance	Peak 12–48 hours	(8, 27)
Citrullinated Proteins	Citrullinated Histone H3 (CitH3)	Plasma/Serum	Western blot, ELISA	Highly specific NET marker; rises within 24 hours; associated with stroke severity (NIHSS)	Peak 6–72 hours	(16, 39)
Cell Death Markers	Extracellular DNA (cfDNA)	Plasma	Fluorometric quantification	Reflects NET formation and general cell death; elevated in large infarcts	Continuous altitude >7 days	(40)
	Nucleosomes	Plasma/Serum	ELISA	NET degradation products; elevated particularly in cardioembolic stroke	Peak 24–72 hours	(41)
NET Proteases	Neutrophil Elastase (NE)	Plasma/Serum	ELISA, activity test	Indicates NET protease activity; associated with blood-brain barrier disruption and edema	Peak 12–48 hours	(42)
DAMPs	HMGB1	Plasma/Serum	ELISA, Western blot	Damage signal activating microglia; predicts secondary neuroinflammation	Two-phase: 6 hours and 3–7 days	(43)
Blood biomarkers: microglia activation						
Microglial Markers	Soluble TREM2 (sTREM2)	Plasma	ELISA	Shows microglia activation; elevated in both IS and ICH; associated with hematoma resolution	Peak 3–7 days	(30, 46)
Inflammatory Cytokines	IL-1β, IL-6, TNF-α	Plasma/Serum	Multiplex immunoassay, ELISA	Reflects anti-inflammatory microglia phenotype; predicts poor outcomes and complications	Peak 12–72 hours	(31, 47)
	IL-18	Plasma/Serum	ELISA	Inflammation activation product; elevated in NET-rich environments	Peak 24–72 hours	(45)
CSF biomarkers						
Microglial Activation	sTREM2	Cerebrospinal fluid	ELISA	CNS-specific microglial activation; highest in SAH and severe IS	Peak 3–7 days	(30)
NET Markers in CNS	H3Cit-DNA	Cerebrospinal fluid	ELISA, immunofluorescence	Indicates NET formation within the CNS; particularly high in SAH	Peak 24–72 hours	(48)
BBB Disruption	MMP-9	Cerebrospinal fluid	Zymography, ELISA	Predicts hemorrhagic transformation; associated with NET burden	Peak 12–48 hours	(49)
Neuronal Damage	S100B, NSE	Cerebrospinal fluid	ELISA	Associated with infarct size and neurological deficit	Peak 24–72 hours	(50)
Chronic Inflammation	YKL-40 (CHI3L1)	Cerebrospinal fluid	ELISA	Astrocyte/microglia marker; indicates chronic neuroinflammation	Elevated >7 days	(51)
Thrombus analysis (from thrombus)						
NET Content	H3Cit, MPO, NE immunostaining	Thrombus specimen	Immunohistochemistry	High NET content predicts poor recanalization, increased thrombectomy risk, and worse outcomes	At thrombectomy	(7–9)
Cellular Composition	Neutrophil count, activation markers	Thrombus specimen	Flow cytometry, immunofluorescence	Neutrophil-rich thrombosis is associated with cardioembolic etiology and treatment resistance.	At thrombectomy	(16)
Imaging modalities						
Microglial Activation PET	TSPO tracers (11C-PK11195, 18F-DPA-714)	*In vivo* brain imaging	PET	Visualizes microglia activation from days to weeks after stroke; associated with functional recovery	Days 3-30	(52, 53)
NET Imaging (Experimental)	MPO PET tracers (64Cu-MPO)	*In vivo* brain imaging	PET	Direct visualization of NET load; early detection of thromboinflammation	Experimental	(54)
BBB Permeability	Gadolinium-enhanced DCE-MRI	*In vivo* brain imaging	Dynamic contrast-enhanced MRI	Quantitates blood-brain barrier disruption; clinical standard for edema assessment	Acute to subacute	(55)
Microvascular Perfusion	CT/MR perfusion imaging	*In vivo* brain imaging	Perfusion-weighted imaging	Detects arrest of microvascular blood flow; guides thrombectomy decisions	Acute (<24 hours)	(56)
Future/emerging approaches						
Multimodal Integration	Combined biomarker panels + imaging	Various sources	AI/machine learning algorithms	Personalized risk stratification and treatment selection based on inflammation profiles	Under development	(57)
Real-Time Monitoring	Intraoperative NET imaging	During thrombectomy	Fluorescence/optical imaging	Guiding treatment optimization and predicting complications	Preclinical	(58)
NET Specific Monitors	H3Cit targeted PET ligands	*In vivo* brain imaging	PET	Direct and specific visualization of NET load in the brain	Under development	(59)

## 10 Challenges and future directions

Despite rapid advances in understanding the interactions between NETs and microglia/macrophages in stroke, significant translational hurdles remain. These challenges include biological complexity, treatment timing, drug administration limitations, biomarker reliability, and the design of sensitive clinical trials. Addressing these issues will be essential for the successful integration of NET-targeted and microglia-focused interventions into acute and subacute stroke care.

### 10.1 Temporal windows and treatment sequencing

One of the main obstacles to clinical translation is the rapid temporal evolution of NET formation and microglial activation. During the hyperacute phase (0–6 hours), NETosis begins in the occluded region, while microglia transition from a surveillance state to an activated state; therefore, interventions aimed at suppressing early NETosis or NLRP3 activation may prevent the first wave of BBB breakdown. During the acute phase (6–72 hours), NET load peaks and microglia exhibit strong inflammasome activity; this window is likely ideal for DNase I, PAD4 inhibitors, and NLRP3 blockade (4, 50). In the subacute and chronic stages (days to weeks), residual NET fragments impede angiogenesis and glial repair, and therapeutic emphasis may shift to TREM2 agonists, CSF1R modulators, and microglial reprogramming strategies (14, 30).

Adapting treatments to these evolving stages is challenging because clinical workflows rarely include tools for real-time assessment of NET burden or microglial status. Therefore, the development of validated circulating biomarkers and imaging results will be critical to guide the timing of NET and microglia-targeted interventions.

### 10.2 Cell type and compartment-specific targeting

Targeting NETs and microglia raises significant specificity concerns. Systemic PAD4 inhibition can compromise neutrophil function in other organs, while DNase I treatment carries the risk of excessive degradation of extracellular DNA involved in repair processes. Similarly, broad microglial inhibition can impair essential functions such as phagocytosis, synaptic maintenance, and hematoma clearance. Single-cell and spatial transcriptomic studies reveal striking heterogeneity of microglial phenotypes across infarct cores, penumbra, perihematomal regions, and meninges (51). Therefore, future therapies will need to selectively target detrimental microglial states without disrupting neuroprotective or reparative populations. Experimental strategies include EV-mediated delivery of siRNA or PAD4 inhibitors to peri-infarct microglia, microglia-selective NLRP3 inhibitors, and thrombus-directed DNase formulations (70, 71); all are still in early stages of development.

### 10.3 Balancing inflammatory suppression with host defense

NETs and microglial activation are integral components of host defense against infection and hematoma and debris clearance. Therefore, excessive suppression of these pathways may increase the risk of pneumonia or meningitis, impair hematoma resolution in ICH, or delay the removal of necrotic tissue. For example, extensive neutrophil depletion improves outcomes in some experimental models but is not clinically acceptable due to the risk of infection (39, 65). Similarly, CSF1R inhibition may impair debris clearance after intracerebral hemorrhage while reducing inflammatory microglia (12). These concerns support selective modulation of defined pathways such as PAD4, NLRP3, or TLR9 rather than general immunosuppression.

### 10.4 Heterogeneity of stroke subtypes and patient populations

NET burden and microglial phenotypes vary greatly depending on:

• stroke subtype (IS, ICH, or SAH)
• clot etiology (cardioembolic, atherosclerotic, cryptogenic)
• patient age and comorbidities
• reperfusion therapies
• hematoma volume and location

For example, cardioembolic thrombi have higher NET content than large artery atherosclerotic thrombi, while SAH patients exhibit strong NET-complement interactions associated with vasospasm (9, 16, 42).

Personalized approaches will require classification tools based on biomarkers, thrombus analysis, or imaging.

### 10.5 Drug delivery challenges across the BBB

Drug delivery across the BBB remains a significant obstacle. Limited penetration of biologic agents such as DNase I or therapeutic antibodies, rapid systemic clearance of nucleases, difficulty achieving adequate concentrations in microvascular NET regions, and off-target enzymatic degradation in blood limit efficacy. Approaches being investigated include nanoparticle- or EV-based delivery systems, intrathecal or intraarterial administration, and BBB-permeable small molecule NLRP3 inhibitors, all of which require further safety and efficacy testing (38, 47).

### 10.6 Robust biomarker validation and standardization

Circulating NET biomarkers (H3Cit–DNA, MPO–DNA) hold promise. However, lack of test standardization across centers, variable half-lives of biomarkers, impact of concomitant inflammatory conditions and uncertainty in defining clinically meaningful thresholds remain challenges. Similarly, microglia-derived markers (sTREM2, cytokines) require rigorous validation before integration into clinical algorithms (51, 58, 59).

Prospective studies linking NET burden to treatment response are needed.

### 10.7 Designing NET- and microglia-focused clinical trials

Interpreting preclinical findings requires well-designed studies that consider optimal treatment timing (hyperacute, acute, or subacute), biomarker-based patient selection, combination approaches (e.g., DNase I + NLRP3 inhibitor), endpoints reflecting microvascular reperfusion and BBB integrity, and safety assessments for immunosuppression. To date, no phase III trials have directly evaluated NET-targeted therapies in stroke, indicating a critical translational gap (51, 72).

### 10.8 Advancing precision medicine with emerging technologies

High-resolution mapping of NET-microglia niches can identify microdomains of intense crosstalk, cell-type-specific druggable targets, and temporal phenotypic transitions (51).

Moreover, AI-based (especially machine learning) models combining clinical variables, imaging features, and NET biomarkers show promise in predicting tPA failure, thrombus NET burden, risk of hemorrhagic transformation and microglial activation trajectories (7, 39).

Additionally, novel PET tracers for MPO, histones, and extracellular DNA enable real-time assessment of NET burden, and this could guide treatment timing.

### 10.9 Integrated future outlook

Progress in understanding NET-microglia/macrophage interactions highlights several common priorities for translational research. First, effective treatments will likely require combination and sequential approaches, as suppressing NET formation alone or regulating microglial activation in isolation only partially interrupts the self-perpetuating immunothrombotic cycle. Rational pairing (such as early DNase I or PAD4 inhibition to reduce intravascular NET burden, followed by delayed TREM2 agonism, CSF1R modulation, or inflammasome blockade) may better adapt to the changing immune environment from hyperacute injury to subacute repair.

Second, temporal precision will be critical. Early NET-focused strategies are most effective before widespread BBB disruption, whereas microglial-targeted interventions may be more appropriate in the subacute phase, where maladaptive microglial phenotypes predominate. Tailoring treatment timing to the dynamic biology of ischemic and hemorrhagic stroke will require prospective tools that report NET amplitudes, microglial activation states, and neurovascular unit integrity in real time.

Third, biomarker-guided patient selection is expected to play a central role. Circulating NET biomarkers (H3Cit-DNA, MPO-DNA), microglial activation markers (sTREM2, YKL-40), and thrombus NET burden provide complementary indicators of intravascular and parenchymal inflammation. These markers may help identify patients most likely to benefit from NET degradation, PAD4 inhibition, inflammasome blockade, or microglial reprogramming, and may also identify individuals at risk for hemorrhagic transformation or DCI.

Fourth, advances in multimodal imaging, such as TSPO-PET for microglial activation, MPO-targeted PET for neutrophil/NET activity, DCE-MRI for BBB permeability, and CT/MR perfusion for microvascular flow, offer noninvasive methods for monitoring therapeutic response and disease progression. Integrated imaging may ultimately identify spatially restricted inflammatory niches that cannot be detected by blood-based analyses alone.

Finally, AI-powered clinical decision support offers a promising platform for integrating clinical variables, imaging, thrombus characteristics, and biomarker profiles into personalized risk predictions. Provided multicenter datasets, transparent reporting standards, external validation, and regulatory oversight, such models can quantify NET burden, infer microglial activation states, and recommend optimal treatment windows.

Together, these advances suggest that the next phase of stroke immunotherapy will rely on mechanism-based, temporally adaptive, and biomarker-driven strategies that target both NETs and microglia/macrophages. By integrating these principles into study design and clinical workflows, the field can move toward truly personalized modulation of the NET-microglia axis in ischemic and hemorrhagic stroke.

## 11 Conclusions

Neutrophil extracellular traps (NETs) and microglia/macrophages form a tightly interconnected inflammatory axis that links intravascular thrombosis to parenchymal neuroinflammation in ischemic and hemorrhagic strokes. Rather than acting independently, these innate immune components reinforce each other through reciprocal signaling, driving thromboinflammation, microvascular dysfunction, blood-brain barrier disruption, and secondary neuronal damage.

Experimental and clinical data now position this NET-microglia axis as a central determinant of stroke evolution and the source of numerous tractable nodes, including NET formation and clearance, inflammasome and pattern recognition pathways, and microglial phenotype switching. The key challenge will be to modulate these pathways in a phase- and compartment-specific manner that protects host defense, hematoma clearance, and neurovascular repair.

Going forward, the most impactful advances are likely to come from mechanistically informed and carefully timed interventions that incorporate NET and microglial data into stroke research and trial design. By integrating this integrated perspective on thrombinflammation into future studies, the field may ultimately arrive at safer and more effective immunomodulatory strategies that improve functional outcomes across stroke subtypes.
